# Diagnostic Improvements of Deep Learning–Based Image Reconstruction for Assessing Calcification-Related Obstructive Coronary Artery Disease

**DOI:** 10.3389/fcvm.2021.758793

**Published:** 2021-11-03

**Authors:** Yan Yi, Cheng Xu, Min Xu, Jing Yan, Yan-Yu Li, Jian Wang, Si-Jie Yang, Yu-Bo Guo, Yun Wang, Yu-Mei Li, Zheng-Yu Jin, Yi-Ning Wang

**Affiliations:** ^1^State Key Laboratory of Complex Severe and Rare Diseases, Department of Radiology, Peking Union Medical College Hospital, Chinese Academy of Medical Sciences and Peking Union Medical College, Beijing, China; ^2^Canon Medical System, Beijing, China; ^3^Medical Science Research Center, Peking Union Medical College Hospital, Beijing, China

**Keywords:** deep learning, subtraction technique, computed tomography angiography, vascular calcification, coronary artery disease

## Abstract

**Objectives:** The objective of this study was to explore the diagnostic value of deep learning-based image reconstruction (DLR) and hybrid iterative reconstruction (HIR) for calcification-related obstructive coronary artery disease (CAD) evaluation by using coronary CT angiography (CCTA) images and subtraction CCTA images.

**Methods:** Forty-two consecutive patients with known or suspected coronary artery disease who underwent coronary CTA on a 320-row CT scanner and subsequent invasive coronary angiography (ICA), which was used as the reference standard, were enrolled. The DLR and HIR images were reconstructed as CTA_DLR_ and CTA_HIR_, and, based on which, the corresponding subtraction CCTA images were established as CTA_sDLR_ and CTA_sHIR_, respectively. Qualitative images quality comparison was performed by using a Likert 4 stage score, and quantitative images quality parameters, including image noise, signal-to-noise ratio, and contrast-to-noise ratio were calculated. Diagnostic performance on the lesion level was assessed and compared among the four CCTA approaches (CTA_DLR_, CTA_HIR_, CTA_sDLR_, and CTA_sHIR_).

**Results:** There were 166 lesions of 86 vessels in 42 patients (32 men and 10 women; 62.9 ± 9.3 years) finally enrolled for analysis. The qualitative and quantitative image qualities of CTA_sDLR_ and CTA_DLR_ were superior to those of CTA_sHIR_ and CTA_HIR_, respectively. The diagnostic accuracies of CTA_sDLR_, CTA_DLR_, CTA_sHIR_, and CTA_HIR_ to identify calcification-related obstructive diameter stenosis were 83.73%, 69.28%, 75.30%, and 65.66%, respectively. The false-positive rates of CTA_sDLR_, CTA_DLR_, CTA_sHIR_, and CTA_HIR_ for luminal diameter stenosis ≥50% were 15%, 31%, 24%, and 34%, respectively. The sensitivity and the specificity to identify ≥50% luminal diameter stenosis was 90.91% and 83.23% for CTA_sDLR_.

**Conclusion:** Our study showed that deep learning–based image reconstruction could improve the image quality of CCTA images and diagnostic performance for calcification-related obstructive CAD, especially when combined with subtraction technique.

## Introduction

With excellent sensitivity and negative predictive value, coronary CT angiography (CCTA) has developed into one of the first choices of non-invasive diagnostic strategies for the evaluation of coronary artery disease (CAD) during clinical practice (1–4). However, on account of the blooming and beam hardening artifacts, the accuracy of stenosis evaluation related to calcified plaques was still unsatisfactory, since the calcification might lead to overestimation of the stenosis and excessive downstream testing (5–7).

Deep learning–based image reconstruction (DLR) has been demonstrated as potentially further valuable for improving image quality and reducing dose for CCTA images (8, 9). In addition, subtraction CCTA approaches have been investigated previously and put forward in decreasing the impacts of the calcification artifacts, improving the image quality (10) and diagnostic accuracy (6, 11–15). Both Guo et al. (10) and Xu et al. (15) reported that, in comparison with conventional CCTA, subtraction CCTA based on standard kernel iterative reconstruction would allow stenosis regarding and improve the diagnostic accuracy in coronary segments with severe calcification. Takamura et al. (11) also showed the diagnostic ability of subtraction CCTA using the low-radiation dose protocol for patients with calcification was superior to that of conventional CCTA alone.

However, the incremental diagnostic value of the DLR technique combined with subtraction CCTA image has not been fully explored yet. Therefore, we conducted this study to investigate the images quality and diagnostic value of the DLR approach combined with subtraction CCTA images in the evaluation of coronary artery stenosis that is caused by calcified lesions.

## Materials and Methods

### Patient Population

**Figure 1:**
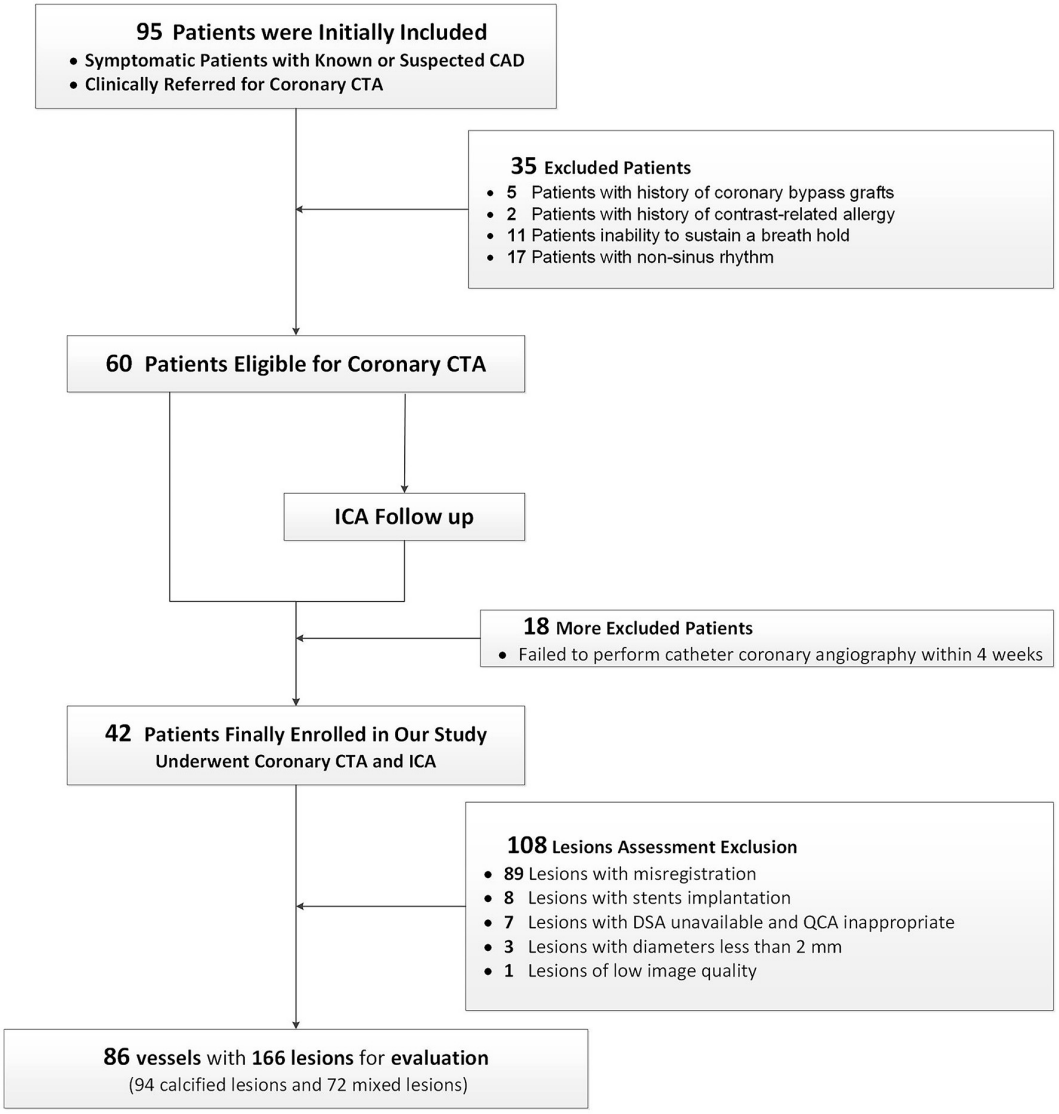
A flow chart. A flow chart of subject enrollment and study design. Based on the predefined inclusion and exclusion criteria, 42 patients successfully underwent CCTA and ICA. CAD, coronary artery disease; CCTA, coronary CT angiography; ICA, invasive coronary angiography.

Consecutive patients with known or suspected coronary artery disease, who underwent coronary CTA and were scheduled for ICA within the next 1 month between March 2020 and April 2021, were initially included. ICA was performed due to a comprehensive situation and the clinical needs of a patient. Exclusion criteria were (1) history of contrast-related allergy; (2) impaired liver or renal function; (3) inability to sustain a breath-hold; (4) pregnancy; (5) non-sinus rhythm; (6) history of coronary bypass graft surgery. Figure 1 shows the patient inclusion flowchart.

Written informed consent was obtained from all the patients, and the study protocol was approved by the institutional ethical committee.

### Coronary CTA Image Data Acquisition

The patients were scanned using a 320-row detector CT scanner (Aquilion ONE GENESIS Edition; Canon Medical Systems Corp, Japan). Sublingual nitroglycerin (1. mg) was used 1–2 min before the scan. β blockers were administrated before the examination. Using a two-breath-hold protocol, the whole scans consisted of a non-contrast scan (2–3 s), followed by a contrast-enhanced scan (5–8 s). In all the patients, the prospective one-beat CTA mode was used with the cardiac phase for scanning a set to 70–80% of the R-R interval (heart rate <75 bpm) or to 35–55% (heart rate >75 bpm). Iodinated contrast media (370 mg I/ml) was administered via a 20 G trocar in the antecubital vein by a dual-syringe power injector (DUAL SHOT GX, Nemoto-Kyorindo, Tokyo) with a protocol based on the BMI of the patients individually. The contrast agent volume was injected at a rate of body weight (kg) ×0.053 ml/s in 10 s (fixed), followed by 30-ml saline at the same injection rate. A bolus-tracking technique was used with a region of interest (ROI) placed in the descending aorta (attenuation threshold: 280 HU), and the scan started automatically with a delay time of 10 s. The scan ranged from the carina to the level of the diaphragm to include the entire heart. Other acquisition parameters were as follows: tube voltage, 100 kVp; gantry rotation time, 275 ms: z-coverage, 120–160 mm; collimation, 320 × 0.5 mm. The tube current was adjusted automatically with noise index *SD* = 33.

### CT Image Reconstruction

The initial CCTA image data were reconstructed with Hybird-IR [Adaptive Iterative Dose Reduction (AIDR) 3D, Cardiac kernel, FC09] and DLR [Advanced Intelligent Clear-IQ Engine (AiCE), Cardiac kernel]. All reconstructions had a slice thickness of 0.5 mm with 0.5 mm intervals. Afterward, these two groups of images with their corresponding non-contrast images were sent to a dedicated post-processing software to obtain subtraction CCTA (^SURE^Subtraction Canon Medical Systems, Otawara, Japan). The subtraction algorithm was based on the combination of global non-rigid registration and local rigid refinement, and the registered non-contrast data set was subtracted from the contrast data set to finally remove calcification (12).

The effective radiation dose was estimated as the dose length product multiplied by a conversion coefficient for the chest (0.026 mSv/mGy/cm) (16), CTDIvol, and DLP of each patient was also recorded. The total dose was calculated for pre-contrast and post-contrast scans.

### Evaluation of Image Quality

The image quality was evaluated on a per-vessel basis and a per-patients basis using the Likert 4 stage score (17). Score 4: non-diagnostic, poor image quality that precluded appropriate evaluation of the coronary arteries due to severe artifacts; score 3: adequate, reduced image quality because of artifacts but sufficient to rule out obstructive CAD; score 2: good, minor artifacts but image quality was adequate for diagnostic evaluation; and score 1: excellent, absence of artifacts and no structural discontinuity. Two radiologists with 5 (CX) and 8 years (YY) of experience in cardiac radiology assessed all images independently. Any discrepancy between the observers was settled by consensus.

The mean attenuation value and noise [defined as the SD of ROI] in the aortic root and adjacent adipose tissue were measured. The mean attenuation value in the proximal segment of the three main coronary arteries [left anterior-descending artery (LAD), left circumflex (LCX), and right coronary artery (RCA)] and the adjacent adipose tissue were also measured. The signal-to-noise ratio (SNR) and contrast-to-noise ratio (CNR) of each part were calculated. The circular ROI should be as large as possible but with care taken to avoid the tube wall and calcification. The calculation formula for SNR and CNR was as follows:


$$\begin{array}{lll} SNR & = & CT{\;}_{lumen}/\;Noise{\;}_{aortic}\\ CNR & = & \left(CT_{\;lumen}-\;CT{\;}_{tissue}\right)/\;Noise_{\;aortic} \end{array}$$

### Diagnostic Performance Evaluation

All the data were transferred to a post-processing workstation for stenosis analysis (Vitrea Workstation, Version 4.0693). Of all the included patients, the calcified or mixed plaques of each segment in the per vessel were selected for obstructive CAD assessment, which was defined as ≥50% luminal stenosis in diameter and area evaluation, respectively. Segments with no plaque, non-calcified plaques, stent implanted, and a diameter <2 mm or low-image quality were initially eliminated.

All the stenosis would be evaluated on a per-lesion basis on each of the four types of images as aforementioned. Parameters, including minimum diameters, maximum diameters, effective diameters, and areas, were measured at the site of the stenosis and the proximal and distal points of the stenosis based on the cross-sectional view of multiplanar reconstruction. For quantitative evaluation, the stenosis degree was calculated using the formula below:


$$\begin{array}{l} Stenosis\;Degree\;=\frac{\begin{array}{c} minimum\;luminal\;measurement\;at\;the\\ \;site\;of\;the\;stenosis \end{array}}{\begin{array}{c} mean\;measurement\;at\;the\;proximal\;and\\ \;distal\;of\;the\;stenosis \end{array}} \end{array}$$

### Invasive Coronary Angiography

**Figure 2:**
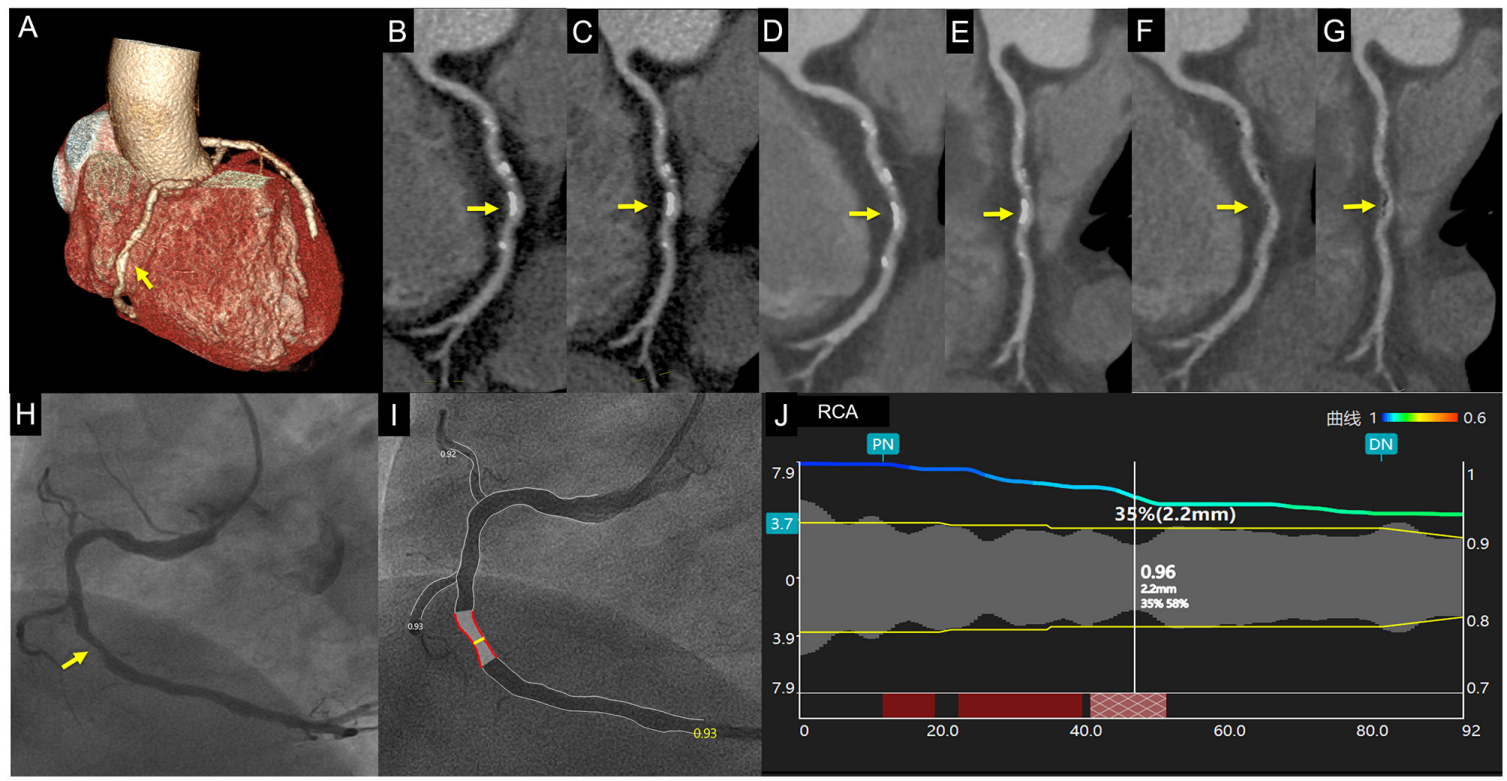
Case 1. A 67-year-old man with chest distress. Volume rendering and curved multiplanar reformation of traditional CTA_HLR_ **(A–C)** images show ~50% stenosis of the middle right coronary artery caused by calcified plaque. CTA_DLR_ **(D,E)** images show superior image quality yet a similar degree of stenosis. CTA_sDLR_ images of corresponding position **(F,G)** show 35% stenosis. Invasive coronary angiography **(H)** and quantitative coronary angiography **(I,J)** confirm the stenosis degree of 35%. Written informed consent was obtained from an individual for the publication of any potentially identifiable images or data included in this article. The yellow arrow shows the location of coronary stenosis.

**Figure 3:**
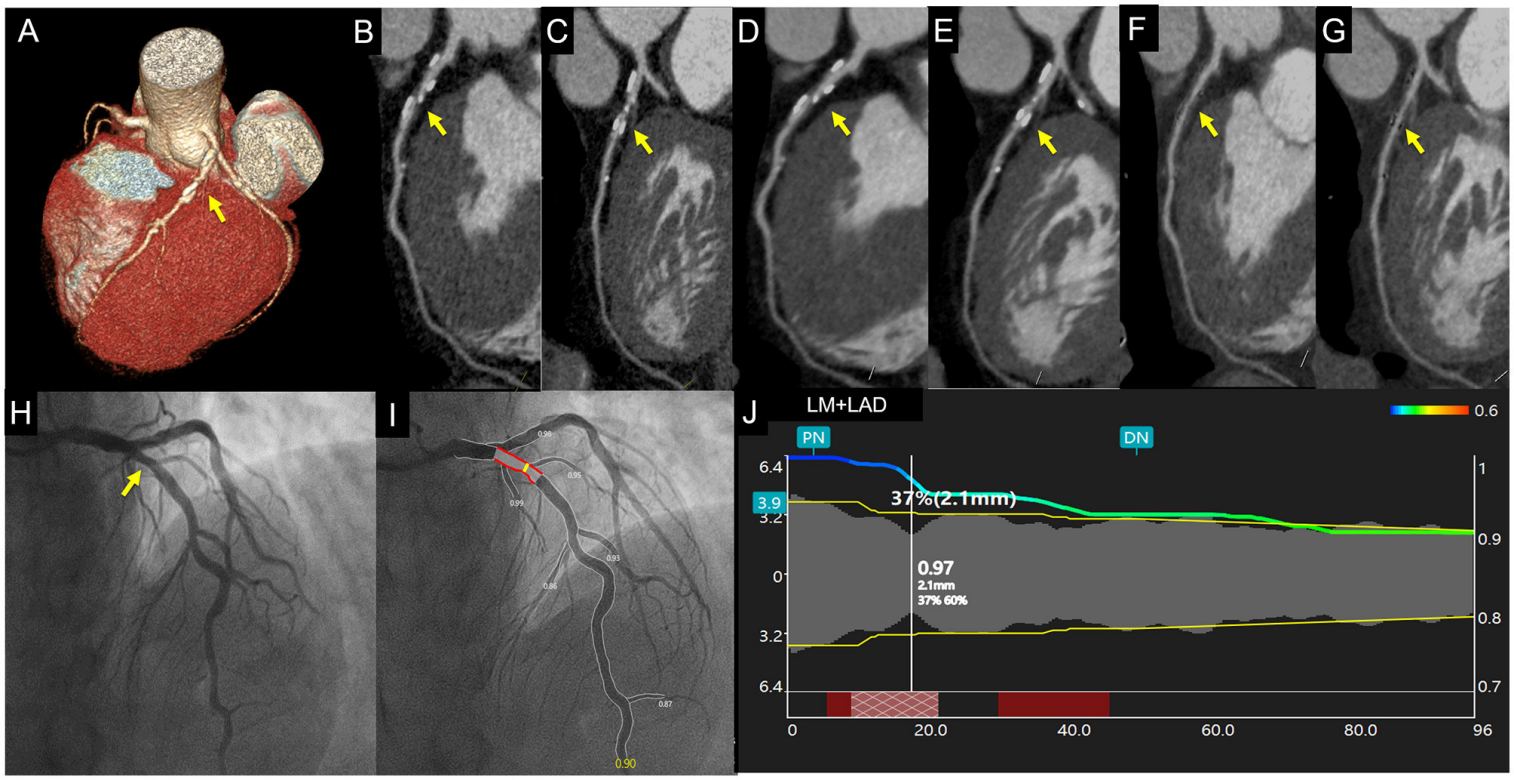
Case 2. A 67-year-old asymptomatic patient. Volume rendering and curved multiplanar reformation of traditional CTA_HLR_ **(A–C)** images show about 65% stenosis of a proximal left anterior descending coronary artery caused by calcified plaque. CTA_DLR_ **(D,E)** images show superior image quality yet a similar degree of stenosis. CTA_sDLR_ images of corresponding position **(F,G)** show mild stenosis (40%). Invasive coronary angiography **(H)** and quantitative coronary angiography **(I,J)** confirm the stenosis degree of 37%. Written informed consent was obtained from the individual for the publication of any potentially identifiable images or data included in this article. The yellow arrow shows the location of coronary stenosis.

Invasive coronary angiography was performed on Allura Xper UNIQ FD10 (Philips Medical Systems, Nederland) using the standard Judkins technique, and images were acquired in multiple projections, where at least two orthogonal projections were obtained in order to assess target vessels. Quantitative coronary angiography (QCA) was performed using dedicated software (AngioPlus Core 1.0, Pulse Medical Imaging Technology, Shanghai, China) by an experienced analyst. Catheter calibration or isocenter calibration was used. Based on the automatically delineated lumen contours, the coronary lumen was reconstructed. Stenosis was then manually selected on a specific segment, and stenosis parameters were quantified automatically (Figures 2, 3).

### Statistical Analysis

Statistical analyses were performed using R software (version 3.6.1, http://www.R-project.org). Quantitative variables were expressed as mean values ± standard deviations, and categorical variables were expressed in terms of frequency and composition ratio (%). Normally distributed continuous variables were evaluated and compared using a paired-student *t*-test, while the Wilcoxon test was used for continuous variables with the abnormal distribution. Image quality and stenosis assessment were compared among all four types of images: CTA_DLR_, CTA_HIR_, CTA_sDLR_, and CTA_sHIR_. Interobserver agreement was assessed by using the kappa coefficient. Sensitivity, specificity, negative predictive value (NPV), positive predictive value (PPV), and diagnostic accuracy for CTA were calculated. *p* < 0.05 was considered statistically significant.

## Results

### Patient Population

Based on the predefined inclusion and exclusion criteria, a total of 42 patients (32 men and 10 women; 62.9 ± 9.3 years) were finally included (Figure 1). The characteristics of the 42 patients are specified in Table 1. Finally, 86 vessels with 166 lesions (92 calcified and 72 mixed lesions) were included for further analysis. The mean radiation dose was 3.7 ± 2.0 mSv.

**Table 1:** Detailed patient baseline characteristics.

Age (year)	62.9 ± 9.3
Male (%)	32 (76%)
Body mass index (kg/m^2^)	25.7 ± 2.9
Diabetes	18 (43%)
Hypertension (%)	30 (71%)
Hypercholesterolemia (%)	31 (34%)
Smoking (%)	30 (71%)
CAD family history (%)	12 (29%)
Previous stent (%)	9 (21%)
Agatston score, median (25–75%)	276.1 (151.9–609.9)
CTDI_vol_ (mGy)	37.6 ± 11.5
DLP (mGy × cm)	145.2 ± 81.2
Effective dose (mSv)	3.7 ± 2.0
Mean heart rate	65.8 ± 8.2
Number of lesions per patient	4.0 ± 2.1
Calcified plaques (%)	133 (80%)
Mixed plaques (%)	33 (20%)

*CAD, coronary artery disease; CTDI_vol_, the volume of CT dose index; DLP, dose length product*.

### Qualitative Assessment of CT Image Quality

Qualitative image quality was evaluated in a total of 42 patients with 86 coronary arteries. On a per-patient basis, the overall average image quality scores of CTA_DLR_ and CTA_HIR_ were 1.07 ± 0.34 and 1.43 ± 0.58 (*p* < 0.001), and the average scores of CTA_sDLR_ and CTA_sHIR_ were 1.10 ± 0.29 and 1.62 ± 0.69 (*p* < 0.001), respectively. On a per-vessel basis, the overall average image quality scores of the three coronary arteries for CTA_DLR_ and CTA_HIR_ were 1.16 ± 0.39 and 1.68 ± 0.71 (*p* < 0.001), and the average scores for CTA_sDLR_ and CTA_sHIR_ were 1.11 ± 0.39 and 1.49 ± 0.59 (*p* < 0.001), respectively.

There was a significant difference between CTA images with DLR reconstruction and HIR reconstruction in both patient-based and vessel-based IQ scores (all *p* < 0.001). Table 2 reports the detailed IQ scores (1–4) for qualitative image quality evaluation.

**Table 2:** Qualitative image quality parameters.

	**Mean**	**Diagnostic segments**			**Non-diagnostic segments**	***P*** **-values**	
		**S1**	**S2**	**S3**	**S4**	**CTA_**HIR**_ vs. CTA_**DLR**_**	**CTA_**sHIR**_ vs. CTA_**sDLR**_**
**Per-vessel basis**							
CTA_HIR_	1.49 ± 0.59	70 (56%)	49 (39%)	6 (5%)	0 (0%)	<0.0001	<0.0001
CTA_DLR_	1.11 ± 0.39	114 (91%)	8 (6%)	3 (2%)	0 (0%)		
CTA_sHIR_	1.68 ± 0.71	57 (46%)	52 (42%)	15 (12%)	1 (1%)		
CTA_sDLR_	1.16 ± 0.39	106 (85%)	18 (14%)	1 (1%)	0 (0%)		
**Per-patient basis**							
CTA_HIR_	1.43 ± 0.58	26 (62%)	14 (33%)	2 (5%)	0 (0%)	<0.0001	<0.0001
CTA_DLR_	1.07 ± 0.34	40 (95%)	1 (2%)	1 (2%)	0 (0%)		
CTA_sHIR_	1.62 ± 0.69	21 (50%)	16 (38%)	5 (12%)	0 (0%)		
CTA_sDLR_	1.10 ± 0.29	38 (90%)	4 (10%)	0 (0%)	0 (0%)		

*DLR, deep learning–based image reconstruction; HIR, hybrid iterative reconstruction; sDLR, subtraction deep learning–based image reconstruction; sHIR, subtraction hybrid iterative reconstruction*.

### Quantitative Assessment of CT Image Quality

There was significant difference in CT attenuation and noise at the aortic root between CTA_DLR_ and CTA_HIR_ (465.66 ± 79.31 vs. 475.50 ± 85.06, *p* = 0.001; 18.00 ± 3.62 vs. 26.56 ± 4.27, *p* < 0.001). Analogously, significant difference in CT attenuation and noise at the aortic root has been shown between CTA_sDLR_ and CTA_sHIR_ (420.25 ± 85.36 vs. 427.10 ± 84.28, *p* = 0.003; 25.25 ± 4.43 vs. 34.23 ± 7.64, *p* < 0.001; Table 3).

**Table 3:** Quantitative image quality parameters.

	**CTA_**HIR**_**	**CTA_**DLR**_**	**CTA_**sHIR**_**	**CTA_**sDLR**_**	***P*** **-values**	
					**CTA_**DLR**_ vs. CTA_**HIR**_**	**CTA_**sDLR**_ vs. CTA_**sHIR**_**
**CT attenuation**						
Ao	475.50 ± 85.06	465.66 ± 79.31	427.10 ± 84.38	420.25 ± 85.36	0.001	0.003
LM	423.77 ± 113.99	426.40 ± 85.10	403.72 ± 85.80	391.60 ± 72.20	0.402	0.147
LAD	375.30 ± 114.38	359.10 ± 108.24	337.93 ± 94.88	319.93 ± 90.84	0.137	0.001
LCX	386.26 ± 88.21	366.80 ± 87.93	346.39 ± 88.41	310.06 ± 109.25	<0.001	0.001
RCA	405.55 ± 75.77	407.07 ± 77.04	380.72 ± 75.84	372.53 ± 77.99	0.441	0.045
**Noise**						
Ao	26.56 ± 4.27	18.00 ± 3.62	34.23 ± 7.64	25.25 ± 4.43	<0.001	<0.001
LM	24.40 ± 8.38	16.15 ± 6.95	27.73 ± 10.19	20.70 ± 9.39	<0.001	<0.001
LAD	24.11 ± 10.18	17.76 ± 8.24	24.90 ± 9.83	19.76 ± 11.30	<0.001	0.001
LCX	21.64 ± 8.41	17.35 ± 8.66	23.98 ± 10.72	19.95 ± 10.17	0.001	0.006
RCA	20.73 ± 8.80	16.13 ± 22.69	26.23 ± 10.21	21.82 ± 9.11	0.170	0.038
**SNR**						
Ao	18.32 ± 4.24	27.24 ± 8.72	13.15 ± 4.07	17.54 ± 7.22	<0.001	<0.001
LM	16.46 ± 4.89	24.83 ± 7.90	12.46 ± 4.11	16.37 ± 6.68	<0.001	<0.001
LAD	14.49 ± 5.14	21.00 ± 8.95	10.39 ± 3.76	13.21 ± 5.95	<0.001	<0.001
LCX	14.0.93 ± 4.39	21.60 ± 8.61	10.72 ± 3.93	12.98 ± 6.38	<0.001	0.002
RCA	15.63 ± 4.00	23.57 ± 6.52	11.59 ± 3.44	15.06 ± 4.66	<0.001	<0.001
**CNR**						
Ao	22.28 ± 4.85	33.20 ± 10.05	16.23 ± 4.58	21.72 ± 8.26	<0.001	<0.001
LM	20.29 ± 5.19	30.45 ± 9.76	15.58 ± 4.83	20.29 ± 7.52	<0.001	<0.001
LAD	18.36 ± 5.60	26.24 ± 8.66	13.76 ± 4.36	17.46 ± 6.75	<0.001	<0.001
LCX	18.92 ± 4.57	27.57 ± 10.08	13.87 ± 4.52	16.71 ± 6.55	<0.001	0.001
RCA	19.55 ± 4.35	29.11 ± 7.52	14.49 ± 4.02	18.98 ± 5.34	<0.001	<0.001

*DLR, deep learning–based image reconstruction; HIR, hybrid iterative reconstruction; sDLR, subtraction deep learning–based image reconstruction; sHIR, subtraction hybrid iterative reconstruction; Ao, aorta root; LM, left main artery; LAD, left anterior descending; LCX, left circumflex; RCA, right coronary artery*.

The SNR values at the aortic root of CTA_DLR_, CTA_HIR_, CTA_sDLR_, and CTA_sHIR_ were 27.24 ± 8.72, 18.32 ± 4.24, 17.54 ± 7.22, and 13.15 ± 4.07, respectively. The CNR values at the aortic root of CTA_DLR_, CTA_HIR_, CTA_sDLR_, and CTA_sHIR_ were 33.20 ± 10.05, 22.28 ± 4.85, 21.72 ± 8.26, and 16.23 ± 4.58, respectively. The SNR and CNR at the aortic root of CTA images with DLR reconstruction were significantly superior to that with HIR reconstruction for both situations with and without subtraction (all *p* < 0.001). Detailed results of the quantitative image analysis are listed in Table 3.

### Diagnostic Performance Evaluation

About 274 calcified or mixed coronary lesions were obtained. One hundred and eight lesions were eliminated: eighty-nine lesions with misregistration, seven lesions with QCA unavailable, three vessels with diameters <2 mm, eight vessels with stent implantation, and one lesion of low-image quality. Finally, 166 calcified or mixed coronary lesions in 86 vessels of 42 patients were analyzed for diagnostic performance. The kappa value of the interobserver agreement was good (kappa = 0.82).

The sensitivity and the specificity to detect area stenosis ≥50% defined by QCA were 74.19% and 85.58% for CTA_sDLR_; 79.03% and 75.96% for CTA_DLR_; 74.19% and 83.65% for CTA_sHIR_; 85.48% and 74.04% for CTA_HIR_, respectively. The sensitivity and the specificity to detect diameter stenosis ≥50% defined by QCA were 90.91% and 83.23% for CTA_sDLR_; 100.00% and 67.10% for CTA_DLR_; 90.91% and 74.19% for CTA_sHIR_, 100.00% and 63.23% for CTA_HIR_, respectively.

**Figure 4:**
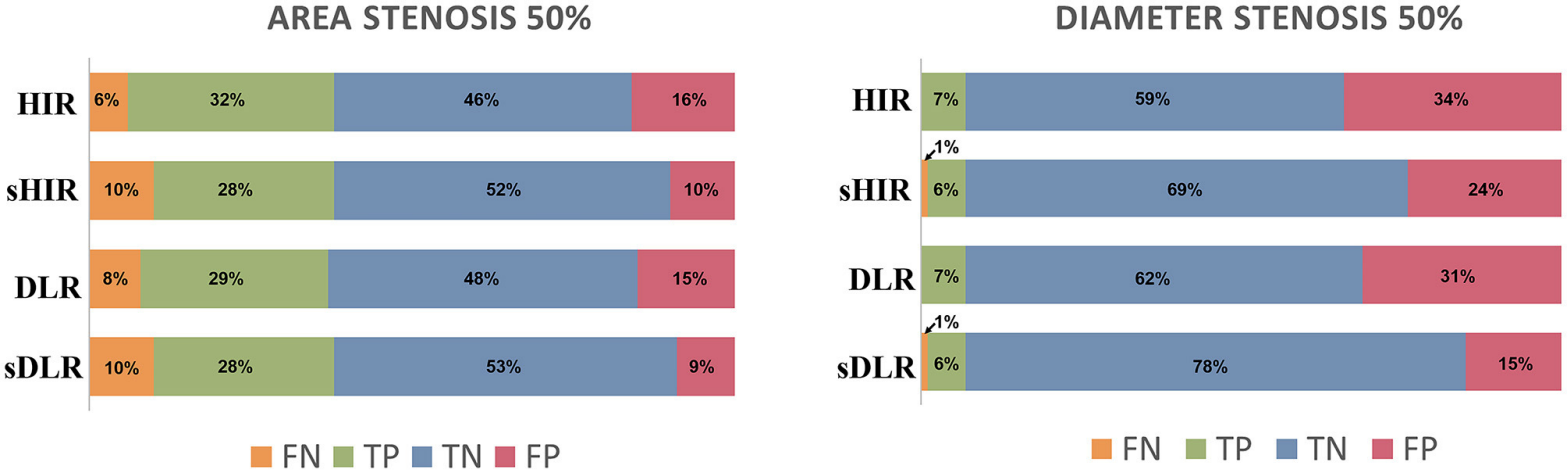
Stenosis evaluation. The false positive rates of CTA_sDLR_ and CTA_sHIR_ for luminal area stenosis ≥50% were 9 and 10%, respectively, while the corresponding values for luminal diameter stenosis ≥50% were 15 and 24%. DLR, deep learning–based image reconstruction; HIR, iterative reconstruction; sDLR, subtraction deep learning–based image reconstruction; sHIR, subtraction iterative reconstruction; TP, true positive; FP, false positive; TN, true negative; FN, false negative.

The false positive rates of CTA_sDLR_, CTA_DLR_, CTA_sHIR_, and CTA_HIR_ for luminal area stenosis ≥50% were 9%, 15%, 10%, and 16%, respectively. And the corresponding false positive rates for luminal diameter stenosis ≥50% were 15%, 31%, 24%, and 34%, respectively (Figure 4).

The diagnostic accuracies of CTA_sDLR_, CTA_DLR_, CTA_sHIR_, and CTA_HIR_ to identify calcification-related obstructive area stenosis and diameter stenosis were 81.33%, 77.11%, 80.12%, and 78.31%, respectively; and 83.73%, 69.28%, 75.30%, and 65.66%, respectively. The CTA_sDLR_ with calcification-related obstructive area stenosis evaluation showed the highest diagnostic performance. Table 4 shows the detailed diagnostic values of the CTA images with different reconstruction techniques.

**Table 4:** Diagnostic accuracy of the CTA images with different reconstruction techniques.

	**FN**	**TP**	**TN**	**FP**	**Sensitivity (%)**	**Specificity (%)**	**PPV (%)**	**NPV (%)**	**Accuracy (%)**
**≥50% area stenosis**									
CTA_HIR_	9 (5.42%)	53 (31.93%)	77 (46.39%)	27 (16.27%)	85.48 (75.78, 93.30)	74.04 (65.01, 81.86)	66.25 (55.22, 75.49)	89.53 (82.07, 96.25)	78.31 (71.41, 84.34)
CTA_sHIR_	16 (9.64%)	46 (27.71%)	87 (52.41%)	17 (10.24%)	74.19 (63.49, 85.29)	83.65 (75.45, 90.19)	73.02 (60.17, 83.99)	84.47 (77.55, 92.00)	80.12 (74.10, 86.14)
CTA_DLR_	13 (7.83%)	49 (29.52%)	79 (47.59%)	25 (15.06%)	79.03 (68.46, 88.52)	75.96 (67.00, 84.47)	66.22 (54.48, 77.08)	85.87 (77.84, 93.44)	77.11 (70.20, 83.73)
CTA_sDLR_	16 (9.64%)	46 (27.71%)	89 (53.61%)	15 (9.04%)	74.19 (62.81, 84.92)	85.58 (78.51, 91.89)	75.41 (62.86, 86.08)	84.76 (77.17, 91.62)	81.33 (74.70, 87.35)
**≥50% diameter stenosis**									
CTA_HIR_	0 (0.00%)	11 (6.63%)	98 (59.04%)	57 (34.34%)	100.00 (100.00, 100.00)	63.23 (55.84, 70.29)	16.18 (7.25, 25.91)	100.00 (100.00, 100.00)	65.66 (57.83, 73.49)
CTA_sHIR_	1 (0.60%)	10 (6.02%)	115 (69.28%)	40 (24.10%)	90.91 (69.64, 100.00)	74.19 (67.08, 80.92)	20.00 (9.91, 32.57)	99.14 (97.29, 100.00)	75.30 (68.07, 81.33)
CTA_DLR_	0 (0.00%)	11 (6.63%)	104 (89.65%)	51 (30.72%)	100.00 (100.00, 100.00)	67.10 (59.74, 74.44)	17.74 (8.20, 27.94)	100.00 (100.00, 100.00)	69.28 (61.47, 75.30)
CTA_sDLR_	1 (0.60%)	10 (6.02%)	129 (77.71%)	26 (15.66%)	90.91 (69.64, 100.00)	83.23 (76.99, 88.96)	27.78 (14.29, 43.82)	99.23 (97.59, 100.00)	83.73 (77.71, 89.16)

*Data are presented as n (%) or values (95% CI). DLR, deep learning–based image reconstruction; HIR, hybrid iterative reconstruction; sDLR, subtraction deep learning–based image reconstruction; sHIR, subtraction hybrid iterative reconstruction; TP, true positive; FP, false positive; TN, true negative; FN, false negative; PPV, positive predictive value; NPV, negative predictive value*.

In the patient-based analysis, ICA identified 30 patients with significant stenosis (positive) and 12 patients without significant stenosis (negative). CTA_HIR_ accurately identified 8 negative patients and 25 positive patients and misjudged 9 patients. CTA_DLR_ accurately identified 11 negative patients and 22 positive patients and misjudged 9 patients. CTA_sDLR_ identified all negative patients and 24 positive patients but misjudged 6 patients. Compare to HIR combined with subtraction technique, additional three patients (33%) were correctly diagnosed using DLR combined with subtraction technique.

## Discussion

This study has two main findings: (1) The image quality of CTA images with deep learning-based reconstruction (DLR) was improved compared to that of CTA images with hybrid iterative reconstruction (IR). (2) DLR technique combined with subtraction CTA images enhanced diagnostic performance for coronary stenosis evaluation of calcified plaques.

The diagnostic performance of traditional coronary computed tomography angiography imaging was limited in the event of calcified plaques, especially for the diffuse or large calcification with intermediate to a high degree of coronary artery calcium score (18, 19). DLR has been developed and utilized in recent years. Previous studies (8, 9) reported that DLR helps in reducing image noise in coronary CTA compared to iterative reconstructions. Our study demonstrated consistent results with that. This can be mainly attributed to higher spatial resolution and better image-noise reduction, which are inherited from the high-quality model-based IR (MBIR) images by means of DLR image training (8, 20).

Besides the image quality improvement with DLR, the diagnostic accuracy could be further increased when coronary subtraction was integrated. Previous studies (15, 21) have concluded diagnostic value enhancement of subtraction CTA in severe calcifications using HIR. Xu et al. (15) showed better diagnostic accuracy and confidence in cases of severe calcification with HIR-based subtraction CCTA information. Vilades Medel et al. (22) reported that subtraction CTA is promising in overcoming the limitations of conventional CTA due to calcium or metal artifacts. In a prospective coronary subtraction multicenter trial, Fuchs et al. (6) further demonstrated that HIR-based subtraction CTA reduced the false-positive rate in well-aligned, calcified segments. The accuracy calculations in target segments without misregistration showed a reduction of the false positives from 72% in conventional CTA to 33% in subtraction CTA.

To our knowledge, this is the first study that evaluated the diagnostic value of DLR combined with subtraction CTA images for calcified-specific stenosis by using ICA as the reference standard. In this study, our results of CTA_sHIR_ for identifying luminal diameter stenosis ≥50% showed a comparative false positive rate of 30%, which was in accordance with that of Fuchs et al. (6). Moreover, we have successfully reduced the false positive rate of CTA_sDLR_ for ≥50% luminal diameter stenosis to 15% (Figures 2, 3). As Fuchs et al. (6) reported, they reduced the false-positive rate in subtraction CTA and came at the expense of 7% false-negative segments, while, in this study, the results showed the corresponding false-negative rate was only 1%. The diagnostic performance of subtraction CCTA is further improved by using DLR with increased specificity (85.58% vs. 74.19%, *p* <0.001) and accuracy (83.73% vs. 75.30%, *p* <0.001). The refined results might be attributed to the enhanced coronary subtraction process with the inputting image quality improvement of the DLR reconstruction approach, i.e., image-noise reduction, blooming artifacts mitigation, and spatial resolution enhancement. In addition, in our study, we found that the false positive rate of CTA_sDLR_ for luminal area stenosis ≥50% has been further reduced to 9%. This may support that the area assessment may also provide valuable information in luminal stenosis evaluation, which could be further analyzed.

The main limitations of this study include the following: First, the sample size of this single-center study was relatively small, it was not capable of detailed analysis based on subtype levels of coronary plaques or gender analysis, and bias might exist since the average calcification degree of patients in this study was not that severe. However, patients with significant coronary stenosis and obstructive CAD might be more likely to be scheduled for ICA directly. Second, to be consistent with the previous studies, the stenosis calculation, including proximal and distal vessels diameters, might carry some limitations since vessel caliber could change abruptly along the vessel course, and we attempted hard to avoid this situation during measurement. Third, subtraction CTA can be challenging due to the periodical heart motion and variation of breath-holding cooperation from the patients; only ones with expected good image quality might be suitable or successful for the subtraction exploration. Although the misregistration artifacts in this study were significantly lower than previously reported (32.5 vs. 50%) (6), further modified schemes, e.g., one-breath protocol or individualization scanning protocol would be needed.

To validate the clinical reliability and reproducibility of DLR combined with subtraction CTA, multicenter prospective trials will be needed. And based on the better imaged quality of DLR subtraction CTA, lower radiation dose protocol, and functional evaluation need to be investigated in subsequent research to further optimize the CCTA performance for patients with obstructive CAD.

In conclusion, the study demonstrated that deep learning–based image reconstruction could improve the image quality of CTA images and diagnostic performance for calcification-related obstructive CAD, especially when combined with subtraction technique.

## Data Availability Statement

The raw data supporting the conclusions of this article will be made available by the authors, without undue reservation.

## Ethics Statement

The studies involving human participants were reviewed and approved by Peking Union Medical College Hospital, Chinese Academy of Medical Sciences and Peking Union Medical College. The patients/participants provided their written informed consent to participate in this study.

## Author Contributions

YY, CX, MX, JY, and Y-NW were contributed to the conception and design, data measurement, and writing the article or revision of the article. Y-YL, S-JY, JW, and Y-BG were contributed to data measurement. YW, Y-ML, and Z-YJ were contributed to study implementation. All authors approved the final manuscript submitted.

## Funding

This research was supported by Beijing Natural Science Foundation (Grant No. Z210013, 2021); the China Postdoctoral Science Foundation (Grant No. 2020T130071, 2020); the CAMS Innovation Fund for Medical Sciences (CIFMS) (Grant No. 2020-I2M-C&T-B-034); the National Natural Science Foundation of China (Grant No. 81873891, 2019); and the Major International (Regional) Joint Research Project of National Natural Science Foundation of China (Grant No. 82020108018, 2021).

## Conflict of Interest

MX, JY, and JW were employed by Canon Medical System (China) Co., Ltd. The remaining authors declare that the research was conducted in the absence of any commercial or financial relationships that could be construed as a potential conflict of interest.

## Publisher's Note

All claims expressed in this article are solely those of the authors and do not necessarily represent those of their affiliated organizations, or those of the publisher, the editors and the reviewers. Any product that may be evaluated in this article, or claim that may be made by its manufacturer, is not guaranteed or endorsed by the publisher.
